# Biofunctional Layered Double Hydroxide Nanohybrids for Cancer Therapy

**DOI:** 10.3390/ma15227977

**Published:** 2022-11-11

**Authors:** Joonghak Lee, Hee Seung Seo, Wooram Park, Chun Gwon Park, Yukwon Jeon, Dae-Hwan Park

**Affiliations:** 1 Department of Engineering Chemistry, College of Engineering, Chungbuk National University, Cheongju 28644, Chungbuk, Republic of Korea; 2 Department of Industrial Cosmetic Science, College of Bio-Health University System, Chungbuk National University, Cheongju 28644, Chungbuk, Republic of Korea; 3 Department of Synchrotron Radiation Science and Technology, College of Bio-Health University System, Chungbuk National University, Cheongju 28644, Chungbuk, Republic of Korea; 4 Department of Biomedical Engineering, SKKU Institute for Convergence, Sungkyunkwan University (SKKU), Suwon 16419, Gyeonggi, Republic of Korea; 5 Department of Intelligent Precision Healthcare Convergence, SKKU Institute for Convergence, Sungkyunkwan University (SKKU), Suwon 16419, Gyeonggi, Republic of Korea; 6 Department of Integrative Biotechnology, College of Biotechnology and Bioengineering, Sungkyunkwan University, Seoburo 2066, Suwon 16419, Gyeonggi, Republic of Korea; 7 Institute of Biotechnology and Bioengineering, College of Biotechnology and Bioengineering, Sungkyunkwan University, Seoburo 2066, Suwon 16419, Gyeonggi, Republic of Korea; 8 Center for Neuroscience Imaging Research, Institute for Basic Science (IBS), Suwon 16419, Gyeonggi, Republic of Korea; 9 Biomedical Institute for Convergence at SKKU (BICS), Sungkyunkwan University, Suwon 16419, Gyeonggi, Republic of Korea; 10 Department of Environmental and Energy Engineering, Yonsei University, Wonju 26493, Gangwondo, Republic of Korea

**Keywords:** layered double hydroxide, nanohybrid, drug delivery system, cancer therapy

## Abstract

Layered double hydroxides (LDHs) with two-dimensional nanostructure are inorganic materials that have attractive advantages such as biocompatibility, facile preparation, and high drug loading capacity for therapeutic bioapplications. Since the intercalation chemistry of DNA molecules into the LDH materials were reported, various LDH nanohybrids have been developed for biomedical drug delivery system. For these reasons, LDHs hybridized with numerous therapeutic agents have a significant role in cancer imaging and therapy with targeting functions. In this review, we summarized the recent advances in the preparation of LDH nanohybrids for cancer therapeutic strategies including gene therapy, chemotherapy, immunotherapy, and combination therapy.

## 1. Introduction

Nanomaterials including layered materials, mesoporous structures, liposomes, micelle, dendrimer, polymeric nanoparticles, and hydrogels have been extensively developed in the fields of biomedical drug delivery systems (DDS) [1,2,3]. As one of the nanomaterials, layered double hydroxides (LDHs) have been intensively researched for various utilization including drugs with therapy, in vivo imaging, in vitro diagnostics, biomaterials, and active implants. In 1842, the inorganic compound with a general formula, Mg_6_Al_2_CO_3_(OH)_16_·4H_2_O, was found and called the name of clay mineral, hydrotalcite [4]. The synthetic method of hydrotalcite-like LDHs having the same chemical composition of Mg and Al was proposed in the laboratory in 1942 using a solution of metal salts and alkali metal hydroxide [5]. Since then, the first report of intercalation of biomolecules into the LDH materials was introduced, and a series of investigations in the delivery system of drugs into cancer cells have led to the new paradigm of biomedical nanomedicine for therapeutic functions [6,7,8,9].

In general, LDHs consist of positively charged brucite-like cationic layers with octahedral structure and unit cell of M(OH)_6_ and negatively charged anionic species inside the interlayer space. Therefore, the chemical formula of LDHs is described as [M^2+^_1-x_ M^3+^_x_(OH)_2_]^x+^ A^n−^_x/n_·mH_2_O, where M^2+^ cations (Mg^2+^, Ca^2+^, Mn^2+^, Co^2+^, Cu^2+^, Zn^2+^, etc.) in the layer are partially substituted by M^3+^ cations (Al^3+^, Cr^3+^, Co^3+^, Ca^3+^, Gd^3+^, etc.) [10,11,12,13,14,15]. As a result of the isomorphic substitution, the host layers generate the positive charges so that negatively charged guests, A^n−^ (Cl^−^, NO_3_^−^, CO_3_^2−^, etc.), are intercalated in the interlayer region to neutralize the charges through electrostatic interaction.

**Figure 1:**
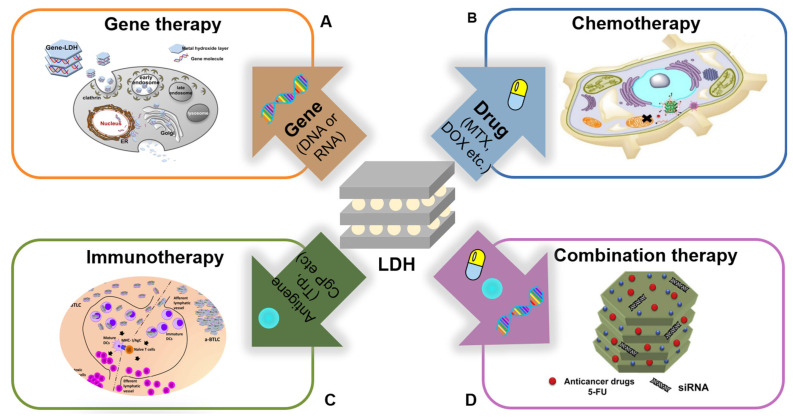
Overview of layered double hydroxide nanohybrids and strategies for therapeutic applications: (**A**) gene therapy; (**B**) chemotherapy; (**C**) immunotherapy; (**D**) combination therapy.

The unique physicochemical properties (large anionic exchange capacity, biocompatibility, and controlled release of biomolecules and drugs in a pH-dependent manner, the hybrid systems), which are based on the LDHs, have also been considered promising inorganic materials for various bioapplications with medical functions [16,17,18,19,20,21,22]. In this review, we summarized the methods to prepare LDH nanohybrids and highlighted the recent advances focusing on four types of therapeutic strategies such as gene therapy, chemotherapy, immunotherapy, and combination therapy as shown in Figure 1 [23,24,25,26,27,28,29,30,31,32,33]. Furthermore, the present progression and the challenge in cancer treatment are discussed for the future development of LDH nanohybrids.

## 2. Preparation Methods for LDH Nanohybrids as Therapeutic Materials

**Figure 2:**
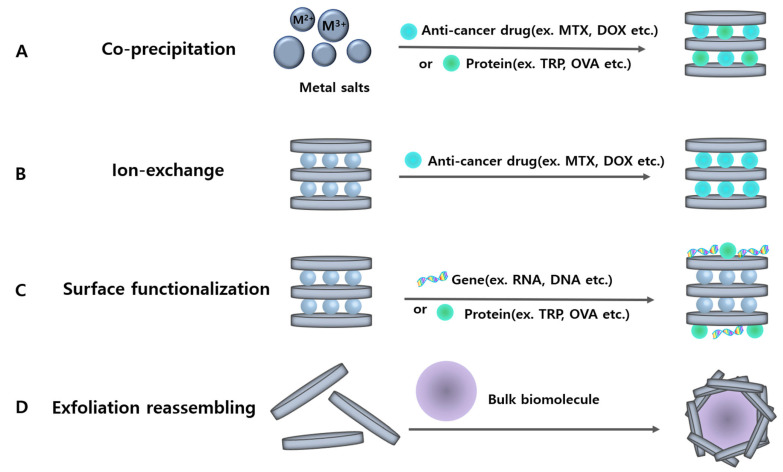
Various ways to prepare LDH nanohybrids for therapeutic application from top to bottom co-precipitation, ion exchange, surface functionalization, and exfoliation reassembling.

For more than two decades, various synthetic methods have been tried to obtain chemically well-defined LDH nanohybrids. In this section, we introduced four approaches for hybridization methods at the nanoscale including co-precipitation, ion exchange, surface functionalization, and exfoliation-reassembling techniques (Figure 2).

The most used method is co-precipitation, which is a simple and cost-efficient way to prepare an intercalation compound [1]. The LDH nanohybrids intercalated with therapeutic agents are commonly realized by base titrating with an aqueous solution of divalent and trivalent metal salts in the existence of desired anionic drugs [14]. This method can be performed at constant or changes in pH, which is determined by the pK_a_ values of functional groups of the therapeutic drug molecules [18]. Additionally, LDHs materials via the co-precipitation method and subsequent hydrothermal treatment have usually regular particle sizes with narrow size distributions and high crystallinity by adjusting conditions of temperature and crystallization time [19]. 

The ion-exchange chemical reaction is also commonly used in methods such as co-precipitation for the synthesis of LDH nanohybrids and can intercalate many different types of anionic biomolecules [12]. Pristine LDHs have exchangeable anions such as NO_3_^−^ or Cl^−^, and an ion-exchange reaction is performed by adding the anions with proper stoichiometric ratio to the pristine LDHs [6]. The whole process is conducted under N_2_ or Ar atmosphere to avoid a contamination reaction with carbonate anions. As a way, the ion-exchange method replaces the pre-occupied anions in pristine LDHs with guest ions that we need for biomedical applications. The guest ions intercalating into LDHs is affected by not only charge distribution and density, but also the size and shape of guest ions [3]. 

When it comes to the gene delivery system, the gene–LDH nanohybrids were prepared not by conventional co-precipitation, but also by simple surface functionalization method. The assembly of negatively charged gene molecules onto the surface of positively charged LDHs can be realized through electrostatic attraction [12]. It is difficult to synthesize artificial genes such as siRNA because of costly utilization. Therefore, surface functionalization would be effective to load gene molecules with low concentrations for a therapeutic window.

The effective method for intercalation or encapsulation of bulky or large biomolecules is the exfoliation-reassembling technique [25]. The LDHs are exfoliated at first in the proper solvent such as formamide. The LDH suspension is then mixed with a solution of biomolecules so that the large-sized molecules can be simultaneously incorporated into the gallery space of LDHs [29]. This exfoliation-reassembling is similar to calcination-reconstruction on the basis of the memory effect of LDHs. Both surface functionalization and exfoliation-reassembling methods would be powerful techniques to incorporate gene molecules into LDHs. 

## 3. Therapeutic Bioapplications with LDH Nanohybrids

Cancer treatment using LDH nanohybrids for bioimaging and therapy has been researched for enhanced clinical methods. In the case of tumor targeting and cancer therapy based on biomedical materials, synergistic combinations of therapy and bioimaging have been applied for the diagnosis and treatment of cancer at once [10,11]. In this section, we focused on selected four cancer treatment approaches including gene-, chemo-, immune-, and combination therapies with various therapeutic agents [34,35,36,37,38,39,40,41].

### 3.1. Gene Therapy

Gene therapy is one of the most promising cancer treatment methods. It is normally difficult to deliver naked gene molecules into target tumors and cancer cells due to low stability, high toxicity, and poor delivery efficiency. In 1999, soluble inorganic LDH material was demonstrated as a gene reservoir for the first time by Choy et al. [23], and then it was realized as a promising gene delivery carrier by showing therapeutic efficacy in vitro experiments [24]. Such intercalation phenomena of genetic molecules including deoxyribonucleic acid (DNA) into LDH materials were theoretically proved by Thyveetil et al. [42]. Since those research results were published, much attention has been paid to the study of efficient gene delivery nanosystems through the bio-LDH nanohybrids with core–shell structures, the manipulation of particle size and/or the anisotropic properties of LDH nanosheets for enhancing the cellular uptake efficiency and reducing the cytotoxicity of LDH nanohybrids as well [28,29].

**Figure 3:**
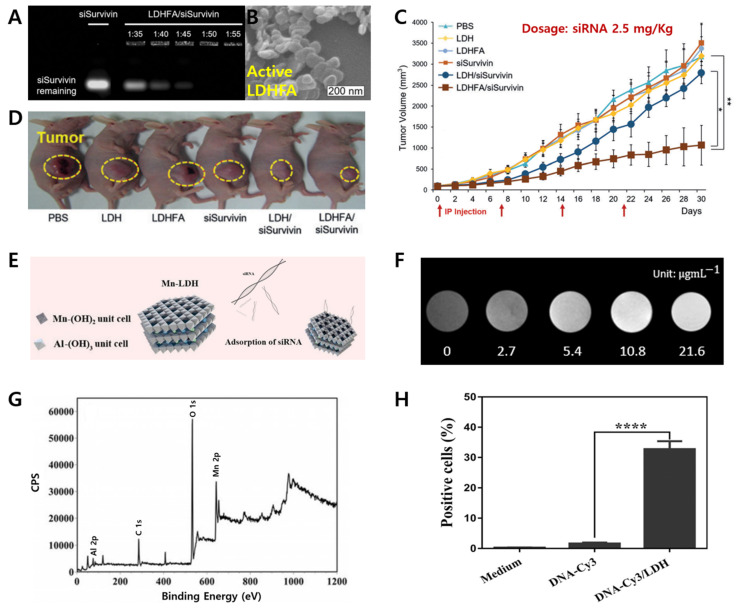
(**A**) Gel retardation assay of siRNA nanohybrids with LDH and LDHFA; (**B**) SEM image of LDHFA/siRNA; (**C**) in vivo tumor growth inhibition rate and (**D**) image of anti-tumor effect of KB tumor-bearing mice treated with LDHFA/siRNA nanohybrids [35] (reproduced from Ref. [35] with permission from the WILEY-VCH Verlag GmbH & Co. KGaA) * *p* < 0.05, ** *p* < 0.01; (**E**) schematic illustration of strategy to synthesize siRNA-Mn-LDH; (**F**) XPS spectra of Mn 2p; (**G**) T1-weighted MR images; (**H**) positive-cell percentage of N2a cells internalizing CD-siRNA/LDH (**** *p* < 0.0001) [44] (reproduced from Ref. [44] with permission from the WILEY-VCH Verlag GmbH & Co. KGaA).

Although many attempts have been made to use LDHs for gene reservoir and cancer therapy, the successful demonstration of gene delivery remained a challenge in in vivo experiments. The In vivo target delivery system for small interference ribonucleic acid (siRNA) using LDH conjugated with folic acid (LDHFA), a cancer-overexpressing receptor-targeting ligand was reported for the first time by Park et al. in 2016 [35]. For the proof-of-principle, various weight ratio of Survivin siRNA (siSurvivin) with either LDH or LDHFA was loaded by a self-assembly process and the biological and chemical stabilities of each nanohybrids are confirmed by gel retardation assay (Figure 3A). The prepared LDHFA/siSurvivin with a particle size of 100 nm completely suppressed the movement since the positively charged LDHFA are spontaneously assembled with negatively charged siRNA through electrostatic attraction (Figure 3B). The reduction in mean sizes and tumor volumes was achieved by up to 66.4% on xenograft mice transfected with KB cells after treatment with the LDHFA/siSurvivin (Figure 3C,D). Such a 3.0-fold higher anti-tumor effect was understood through the tumor-targeting effect by both the clathrin-mediated endocytosis of intrinsic LDH nanoparticles with enhanced permeability and retention (EPR) and folate receptor-mediated endocytosis. With this research, the engineered LDH nanohybrids have fruitfully accelerated the evolution of the next-generation gene delivery system for cancer treatment.

Furthermore, advanced gene delivery with the bioimaging function was proposed through lattice engineering technique and partial replacement of transition metal elements into the LDH layer [43,44]. For example, Manganese-based LDH nanoparticles (Mn-LDH) were reported for gene delivery systems together with bioimaging to obtain not only a therapeutic advantage but also T_1_-weighted magnetic resonance imaging (MRI) (Figure 3E). The successful substitution of divalent Mn element inside the LDH layer was investigated by X-ray photoelectron spectroscopy (XPS) analysis (Figure 3F). It was also confirmed that the Mn-LDH is able to produce a brighter MRI contrast agent for cancer imaging with an *r*_1_ value of around 4.47 mM^−1^s^−1^, which is higher than that of commercial MRI contrast agents on the basis of Gd(III) complexes (Figure 3G). Those Mn-LDH materials also showed low cytotoxicity and therapeutic function as well by delivering the cell-death siRNA (CD-siRNA) with Mn-LDH in order to kill the brain cancer cells more efficiently (Figure 3H), where the high gene delivery efficacy with a simultaneous cancer diagnosis of LDH are proven for dual biofunctional performance. 

Another example of LDH to be highlighted is the nanohybrid of peptide nucleic acid (PNA) for pancreatic ductal adenocarcinoma (PDAC) [45]. The PNA/LDH nanohybrids showed an enhanced reduction rate of tumor size and survival rate of tumor-bearing mice compared with DNA/LDH. Until now, tremendous studies on gene therapy have been performed with several strategies to increase delivery efficiency and therapeutic efficacy. However, limitations still exist on the gene therapy using LDH such as short-term stability of adsorption of gene molecules on the surface of LDHs by self-assembly process, non-specific targeting to be reduced, and/or the cellular fate of LDH, which must be studied on the near future for better efficient gene therapeutic bioapplications.

### 3.2. Chemotherapy

Chemotherapy with LDHs is most often developed and utilized to treat cancer using many different anti-cancer drugs including methotrexate (MTX), doxorubicin (DOX), 5-fluorouracil (5-FU), dacarbazine (DAC), etc. [46,47,48,49]. Chemotherapy is a common powerful cancer treatment approach that uses chemicals to inhibit fast-growing cancer cells in the human body.

Since the MgAl-LDH intercalated with MTX drug was applied to chemotherapy in vitro cell-line experiment in 2004 [26], a series of MTX-LDH nanohybrids have been studied on drug contents, controlled release profile, toxicity, and improvement of colloidal stability as delivery systems. Li et al. reported SiO_2_ dot-coated LDH for MTX carriers and showed effective inhibition in U2OS cell lines compared with MTX-LDH [50]. Furthermore, 5-FU/LDH nanohybrids were utilized with PEGylated hyaluronic acid for targeted drug delivery [51]. In addition to drug delivery, the attempts to apply chemotherapy with bioimaging were made through doping Gd on DOX/LDH-Au [52], coating MnO_2_ on the surface of LDHs [53], using Manganese-iron LDH [54]. 

**Figure 4:**
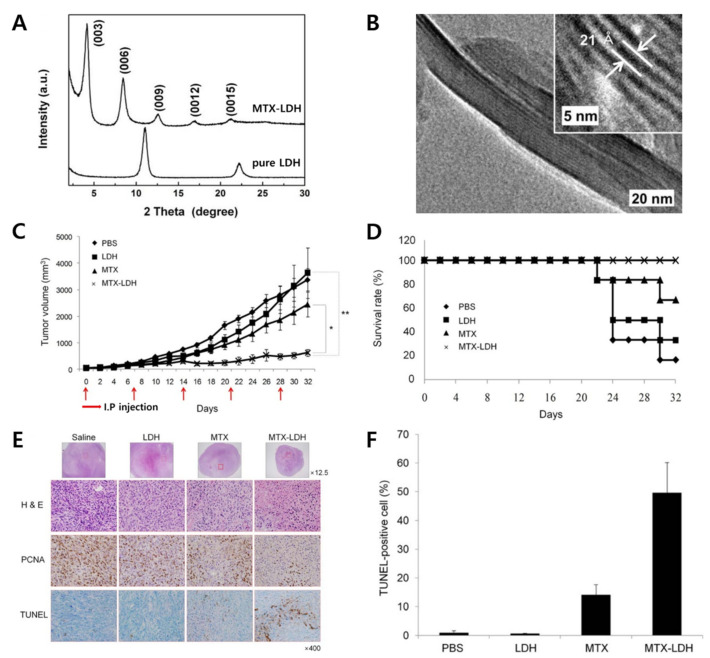
(**A**) X-ray diffraction (XRD) patterns; (**B**) high-resolution transmission electron microscopy (HR-TEM) images of MTX-LDH nanohybrid; (**C**) anti-tumor activity in the MCF7/mot orthotopic breast model * *p* < 0.05, ** *p* < 0.01; (**D**) survival rate of tumor-bearing mice; (**E**) histological and immunohistological analysis of tumor tissue sections from MCF-7/mot orthotopic tumor-bearing mice; (**F**) quantification of TUNEL-positive spots in tumor sections [46] (reproduced from Ref. [46] with permission from the Springer Nature Limited).

For the proof-of-concept of LDH-MTX, the X-ray diffraction pattern is typically investigated at first to confirm a series of (00l) reflective peaks, which indicates accommodation and well-oriented arrangement of MTX drug molecules inside the space of LDH materials (Figure 4A). The image of high-resolution transmission electron microscopy of the MTX-LDH nanohybrids showed that the interlayer distance of the MTX-LDH nanohybrids was approximately 21.0 Å (Figure 4B). The preparation method of MTX-LDH is well described by co-precipitation and subsequent hydrothermal treatment for chemically, structurally, and morphologically controlled production of drug-LDH nanohybrids. 

Very recently, the anti-tumor effect of MTX-LDH was demonstrated in an orthotopic breast cancer model for potential preclinical trials [46]. The tumor growth inhibition was proven by comparing with MTX only, PBS, and LDH to know the efficiency of the therapeutic performance of MTX-LDH. The mean tumor volumes which are treated with the PBS and pristine LDH were 3374.6 mm^3^ and 3638.5 mm^3^ on day 32, respectively. In the case of MTX-LDH, the mean volume of the tumor was determined to be a numerical value of 627.8 mm^3^, whereas the tumor volume treated with MTX only was 2447.6 mm^3^ (Figure 4C). The MTX-LDH nanohybrids also showed an increased survival rate which was associated with tumor growth. The survival rate of the mice injected with MTX-LDH on days 0, 7, 14, 21, and 28 was 100% until day 32. In contrast, the mice treated with PBS, MTX only, and LDH showed survival rates of 16.7%, 33.3%, and 66.6%, respectively (Figure 4D). In the case of the TUNEL assay to analyze induced apoptosis in vivo, few positive spots in tumors were detected in tumors treated with PBS or LDH (Figure 4E). Quantification of the TUNEL-positive spots in tumors treated with MTX-LDH was 49.6%, whereas tumors treated with PBS, LDH, and MTX showed 0.8%, 0.6%, and 14.0% TUNEL-positive spots, respectively.

Due to the limited application for in vivo imaging of the MTX-LDH, certain contrasting agents can be accommodated to trace drug delivery carriers instead of fluorescent dyes. As an example study, single-photon emission computed tomography (SPECT) and positron emission tomography (PET) are applied to LDH nanohybrid for radioisotope (RI)-based tumor imaging. Among RI, Co-57 was utilized for contrasting agents with LDH (Co-57/LDH) [55].

**Figure 5:**
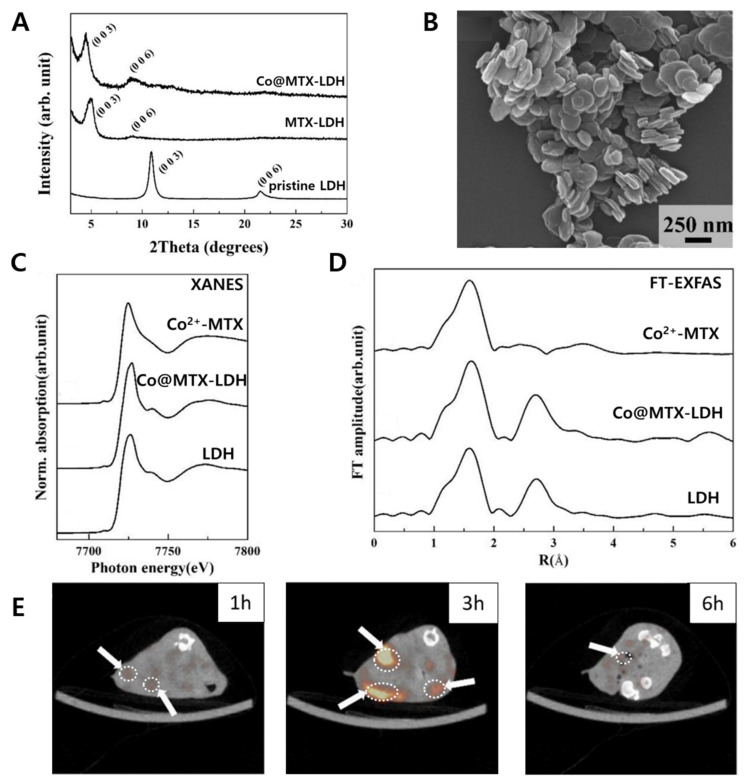
(**A**) X-ray diffraction (XRD) patterns; (**B**) scanning electron-microscopic (SEM) image of the Co@MTX-LDH; (**C**) Co k-edge X-ray adsorption spectroscopy (XAS), (**D**) and Fourier transform-extended X-ray absorption fine structure (FT-EXAFS) spectra; (**E**) SPECT/CT image of a tumor in mouse model after IV injection [31] (reproduced from Ref. [31] with permission from the WILEY-VCH Verlag GmbH & Co. KGaA).

The X-ray diffraction (XRD) demonstrated that the MTX was intercalated into LDH layers. Furthermore, the information that Co^2+^ moiety could increase the (006) peak intensity compared to MTX was demonstrated from the XRD patterns of Co@MTX-LDH (Figure 5A). As shown in Figure 5B, it was clearly confirmed that the particle size and shape of Co@MTX-LDH were around 150 nm with hexagonal plate-like morphology. X-ray absorption spectroscopy (XAS) was studied to confirm the partial substitution of Co^2+^ into the LDH layer of the MTX-LDH nanohybrid. At the Co K-edge, the X-ray absorption near-edge structure (XANES) spectra exhibited that the Co^2+^@MTX-MgAlLDH is similar to the Co^2+^Al^3+^-LDH rather than Co^2+^-MTX complex in accordance with edge position, white-line intensity, and overall spectral shape (Figure 5C). The location incorporated Co^2+^ element into the hydroxide layer of MTX-LDH was observed by Fourier transform extended X-ray absorption fine structure (FT-EXAFS) spectra as well (Figure 5D). The overall spectral shapes of Co@MTX-LDH were also similar to those of CoAl-LDH; however, they were not comparable to that of the Co^2+^-MTX complex in view of the local environment around the Co element.

The time-dependent in vitro cellular uptake of Co-57/LDH was researched in mouse colon carcinoma cell line (CT-57) and human hepatocellular carcinoma cell line (HepG2) by using a γ-counter to measure the radioactivity. The anticancer activity of the Co@MTX-LDH was further suggested indirectly by treating CT-26 cells with MTX-LDH, which strongly proved the substantial apoptosis and cancer-cell suppression after 48 h of incubation (Figure 5E). The bioimaging function of Co@MTX-LDH was clearly evaluated in vivo SPECT/CT images on CT-26 xenografted mouse model, where the SPECT signal after 3 h became stronger than 1 h signal in tumor tissue and then disappeared after 6 h injection by metabolism. As Oh et al. found, the radioisotope-labeled drug-LDH nanohybrids are able to be potential candidates for dual-function biomedical nanomaterials with a high cancer-cell suppression effect and cancer imaging.

**Figure 6:**
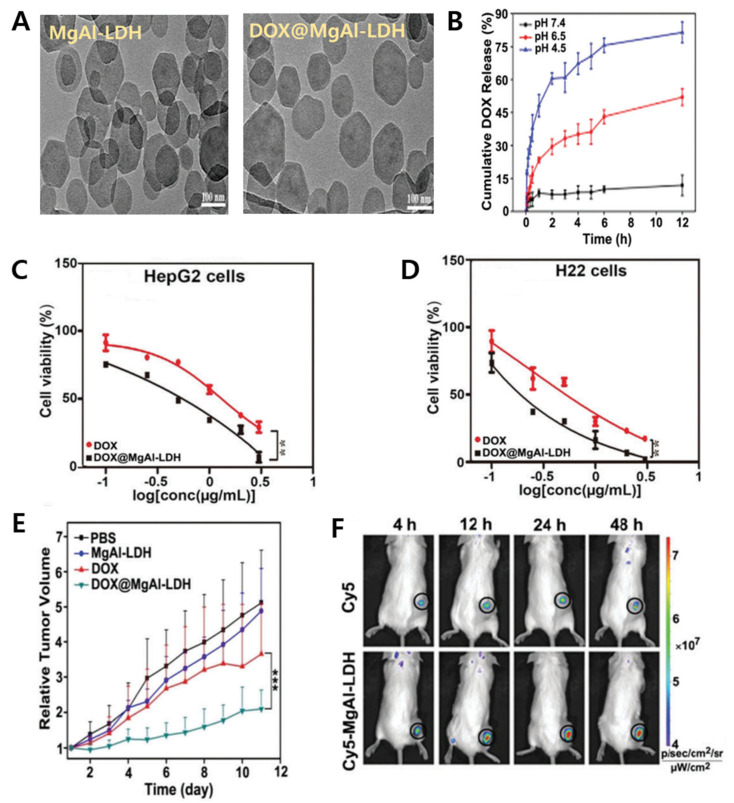
(**A**) TEM images of the MgAl-LDH and DOX@MgAl−LDH; (**B**) release pattern of DOX from the DOX@MgAl−LDH in PBS with different pH values at 37 °C; (**C**) cell viability of the DOX@MgAl−LDH in HepG2 (**D**) and H22 cells; (**E**) tumor volume change in H22 tumor−bearing mice after IV administration of each sample; ** *p* < 0.01, *** *p* < 0.001. (**F**) in vivo Cy5 fluorescence images of H22 tumor−bearing mice at different time courses after IV injection [49] (reproduced from Ref. [49] with permission from the Royal Society of Chemistry).

Other anti-cancer drugs have been applied for chemotherapy as well. The DOX is generally used as a DNA-targeting anti-cancer agent with a positive charge, while MTX and 5-FU are anionic anti-cancer drugs. Such DOX have limits to clinical use since the DOX can cause damage to the heart. To reduce cell toxicity, the DOX drug was intercalated into MgAl-LDH via a base-catalyzed co-precipitation reaction [49]. Typical plate morphology of LDH was observed in both TEM images of MgAl-LDH and DOX@MgAl-LDH (Figure 6A). Additionally, the test of pH-responsive DOX drug release from DOX@MgAl-LDH was performed in PBS buffer with different acidic conditions until 12 h at 37 °C (Figure 6B). After 12 h, the DOX release from the LDH matrix was around 7% at pH 7.4, 46% at pH 6.5 and 80% at pH 4.5, respectively. The DOX@MgAl-LDH had an impact on more effective cell viability than free DOX in both human hepatocarcinoma HepG2 and murine hepatocarcinoma H22 cell lines (Figure 6C,D).

The in vivo anti-cancer activity of prepared DOX@MgAl-LDH was successfully further evaluated in H22 tumor-bearing mice at a DOX dose of 4 mg kg^−1^ once every other day for 5 times (Figure 6E). The DOX@MgAl-LDH demonstrated a significant reduction in tumor growth compared to free DOX. Such inhibition rate of tumor growth of DOX@MgAl-LDH was 64%, whereas free DOX was 36%. Moreover, in vivo biodistribution was investigated to know the biological activity and behavior of LDH nanohybrid. The fluorescence intensity of Cy5-conjugated MgAl-LDH showed much stronger than that of free Cy5 in tumors over time (Figure 6F). The DOX-loaded LDHs have been considered promising efficient chemotherapeutic drug delivery in tumor treatment. 

Moreover, Li et al. recently reported albumin-stabilized LDH (BLDH) which is co-loaded with 5-FU and abraxane (ABX). Therefore, the prepared hybrid gained significant inhibition ability of HCT-116 tumor growth [32]. Such a possibility of a combination of anti-cancer drugs would be a more effective therapeutic application. Another new LDH nanohybrids with polyacrylic acid (PAA) showed a better anti-cancer activity of DOX with confocal microscopy and cellular assays in A549 and osteosarcoma cells (MG-63) [56].

A variety of LDH hybrid systems has still been developed in chemotherapy techniques; however, those LDH nanohybrids must be more studied to prove the safety and efficacy in orthotopic tumor xenografts with metastasis of various tumor types.

### 3.3. Immunotherapy

Cancer immunotherapy differs from other common cancer therapy strategies in its stimulation of the immune system. Immunotherapy targets usually antigens and antigen-presenting cells (APCs) to activate immunogenicity. Therefore, a selection of proper therapeutic agents must be a significant process for immunotherapy to target immune cells such as dendritic cells (DCs), B cells, T cells, macrophages, and natural killer (NK) cells [57,58,59,60]. 

Li et al. synthesized DNA/LDH nanohybrids to activate DCs [61]. They used MgAl-LDH with a ratio of 1 to 1 for the efficient loading of DNA. Their pcDNA3-ovalbumin (OVA)/LDH with CpG nanohybrids showed more effective immunotherapy results than that of CpG-free nanohybrids, more effective inhibition of B16-OVA melanoma tumor growth and higher survival rate of tumor-bearing mice than treated CpG free nanohybrids. 

**Figure 7:**
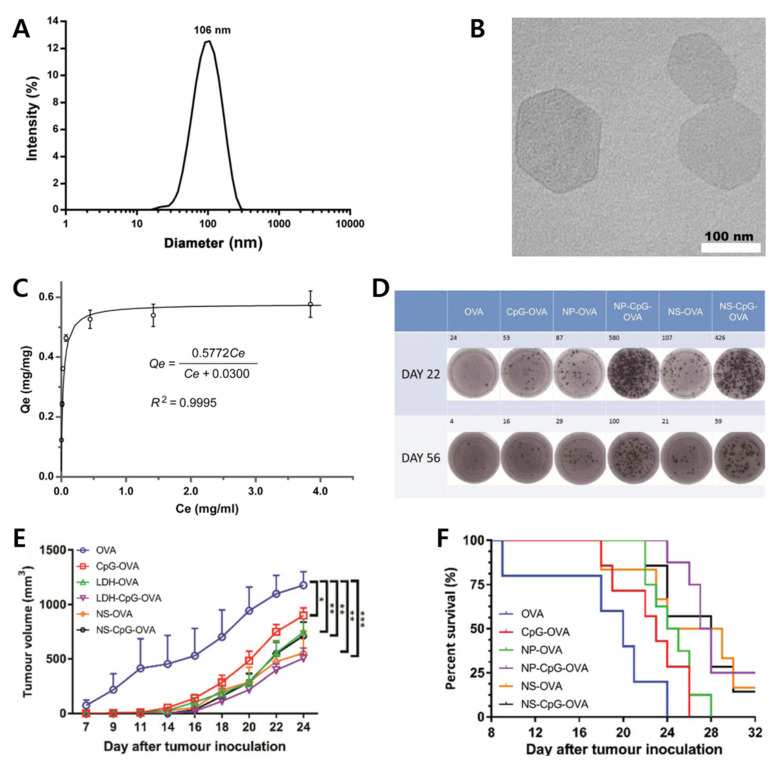
(**A**) Particle size distribution, and (**B**) TEM image of Mg_2_Al-Cl-LDH; (**C**) OVA adsorption isotherm to MgAl-LDH [27] (reproduced from Ref. [27] with permission from Elsevier Ltd.); (**D**) SIINFEKL-specific IFN-γ secretion treated with each sample; (**E**) reduction in tumor growth treated with each sample * *p* < 0.05, ** *p* < 0.01, *** *p* < 0.001. (Two-way ANOVA with Tukey’s post-test); (**F**) percentage of survival mice with EG7-OVA lymphoma cells [63] (reproduced from Ref. [63] with permission from the Royal Society of Chemistry).

The immune systems of the human body make it difficult to efficiently deliver immunotherapeutic agents to immune cells. As one of the studies to solve this problem, Yan et al. have developed an immunotherapy agent by applying effective adjuvants, conjugating Toll-like receptor (TLR) ligand CpG to LDH nanoparticles [62]. EL4 transfection cells (EG7-OVA) were chosen as the tumor model in this research. LDH-based adjuvant-antigen nanohybrid with CpG and OVA (CO-LDH) was prepared by co-precipitation and surface-adsorption. The Mg_2_Al-Cl-LDH displayed approximately 106 nm diameter and hexagonal-shaped morphology with sizes ranging from 40 to 200 nm (Figure 7A,B). A bicinchoninic acid assay was performed to determine the OVA protein adsorption capacity of LDH nanoparticles (Figure 7C). 

The splenocytes were collected from the immunized mice to quantify the antigen-specific cellular response and simulated with either irrelevant peptide or OVA H-2Kb-restricted CTL epitope. The SIINFEKL-specific response of OVA only was weaker than CpG-OVA, NP-OVA, and NS-OVA on day 22 (Figure 7D). The spot number of NP-CpG-OVA and NS-CpG-OVA were increased 4.6- and 3.0-fold compared with those of NP-OVA and NS-OVA, respectively. This is a remarkable improvement in view of NP-CpG-OVA and NS-CpG-OVA cases. The result on day 57 was like day 22. Additionally, the SIINFEKL-specific response of NP-CpG and NS-CpG-OVA were stronger than those of OVA, NP-OVA, NS-OVA, and CpG-OVA. The NP-CpG-OVA showed further inhibition of tumor growth compared with NP-OVA and NS-OVA (Figure 7E). All the tumor-bearing mice treated with OVA only were dead within 24 days while the mice treated with CpG-OVA and NP-OVA were dead within 24.5 and 26.5 days, respectively (Figure 7F). On the other hand, NP-CpG-OVA and NS-CpG-OVA groups survived 27.5 and 28 days, even with two mice and one mouse being alive till the experiment ended, respectively. With this research, both LDH and NSs were proven to be high-potential protein-based antitumor vaccine adjuvants.

**Figure 8:**
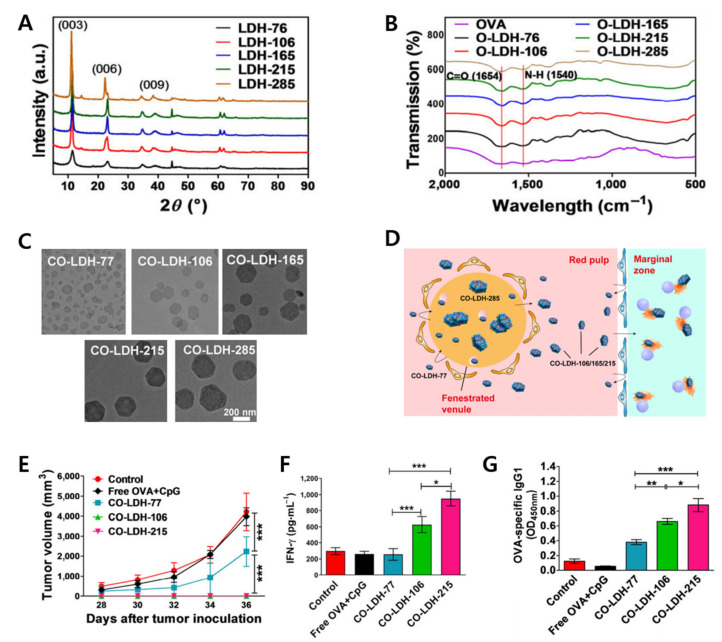
(**A**) XRD patterns of various size of LDH NPs; (**B**) FT-IR spectra of OVA-LDH NPs; (**C**) TEM images of different sizes of OC-LDH; (**D**) schematic illustration of behavior in terms of various sizes of CO-LDH; (**E**) the average volume of tumor after IV vaccinations; (**F**) secreted level of IFN-γ and (**G**) OVA-specific IgG1 (* *p* < 0.05, ** *p* < 0.01, *** *p* < 0.001) [65] (reproduced from Ref. [65] with permission from Springer Nature).

One of the concerning factors for efficient therapy is the size of LDH nanoparticles. Therefore, the determination of the proper size is a critical problem to solve [64]. The effect of the particle size on immunotherapy was recently demonstrated through CpG/OVA-LDH (CO-LDH) [63]. The particle size of LDHs as prepared nanoadjuvants ranged from 77 to 285 nm, and the mouse lymphoma cell line was used in vivo. The XRD patterns of LDH NPs demonstrated characteristic crystal structure (Figure 8A). FT-IR spectra showed C=O (1654 cm^−1^) and N-H (1540 cm^−1^). Therefore, all LDH NPs were hybridized with OVA on their surface (Figure 8B). The morphology of CO-LDHs had a homogeneous shape (Figure 8C). Among the CO-LDH samples with different particle sizes (Figure 8D), the CO-LDH-215 and CO-LDH-106 showed a significant effect on tumor growth inhibition, whereas the CO-LDH-77 was 48% in terms of tumor inhibition (Figure 8E). However, the CO-LDH-215 exhibited more effective evidence in terms of the secreted level of IFN-γ and OVA-specific IgGI for tumor treatment (Figure 8F,G).

Modulating the immunosuppressive tumor microenvironment using microRNA155 (miR155) with LDHs is another strategy for immunotherapy by repolarizing tumor-associated macrophages to the M1 subtype [66]. The miR155-LDH nanohybrids presented enhanced efficiency in the inhibition of tumor growth in TC-1-bearing mice compared with free miR155 and siRNA-LDH. Furthermore, the expression level of STAT3 and ERK1/2 was decreased significantly and NF-κB expression was activated by miR155-LDH nanohybrids. Although immunotherapy is a promising strategy for therapeutic bioapplication, many attempts should be completed to verify how LDH nanohybrids are efficient and safe as compared with other therapies.

### 3.4. Combination Therapy

Although each therapy mentioned above has a significant effect on cancer treatment, there have been many efforts to combine more than two cancer therapies for highly efficient clinical treatment. In this section, attempts to combine therapeutic approaches are highlighted and discussed with different types of anti-cancer agents in LDH nanohybrids. 

**Figure 9:**
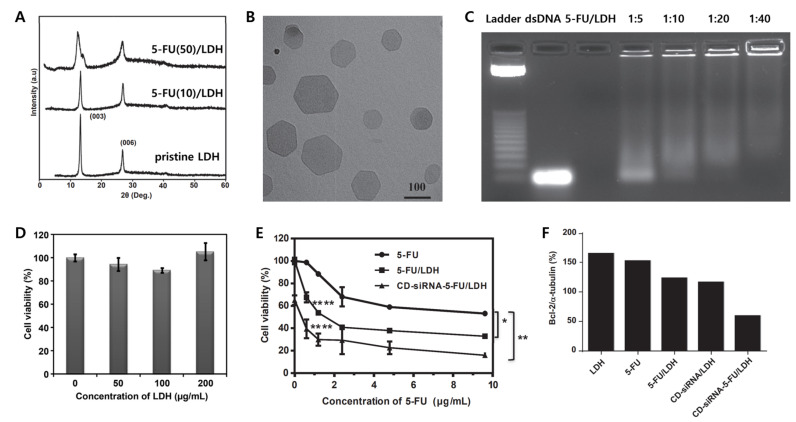
(**A**) XRD patterns of 5-FU/LDH nanohybrid; (**B**) TEM image of the siRNA-5-FU/LDH nanohybrid; (**C**) agarose gel retardation assay with various mass ratios of siRNA:LDH; (**D**) cytotoxicity to various concentration of LDHs to MCF-7 cell lines; (**E**) MTT assay analysis on the viability of MCF-7 cells at the 5-FU concentration ranging from 0 to 9.6 μg/mL and 40 nm concentration of CD-siRNA (* *p* < 0.01, ** *p* < 0.05, **** *p* < 0.0001); (**F**) expression levels of Bcl-2 against α-tubulin. Concentration of CD-siRNA was 2.4 μg/mL and 40 nm, and LDH concentration was 50 μm/mL, respectively [34] (reproduced from Ref. [34] with permission from Elsevier Ltd.).

A representative example of combination therapy is a co-delivery of chemotherapeutic drugs and genes such as 5-FU with Allstars Cell Death siRNA (CD-siRNA) using LDHs [34]. The 5-FU/LDH was synthesized by co-precipitation and ion exchange, the CD-siRNA-5-FU/LDH nanocomplexes were then prepared via the electrostatic interaction between negatively charged siRNAs and positively charged 5-FU/LDH. The XRD patterns of pristine LDH demonstrated (00l) reflections, corresponding to (003) and (006) that revealed the formation of crystalline layered structures. In the case of 5-FU/LDH, (00l) reflection was a weaker and broader peak than pristine LDH because 5-FU was intercalated into the layer of pristine LDH (Figure 9A). The shape of 5-FU/LDH nanohybrids displayed regular hexagonal morphology with sizes ranging from 50 to 150 nm (Figure 9B). From the agarose gel retardation assay, the ability of binding siRNA for 5-FU/LDH was investigated, where 5-FU/LDH was adsorbed effectively with dsDNA when the mass ratio of 5-FU/LDH to dsDNA over 10:1 (Figure 9C).

The cytotoxicity of CD-siRNA-5-FU/LDH was tested in human breast cancer (MCF-7), osteosarcoma (U2OS) and colorectal (HCT-116) cancer cell lines. The cytotoxicity of LDH nanoparticles ranging from 50 to 200 μg/mL to MCF-7 cells revealed cell viability of 90%, even at 500 μg/mL (Figure 9D). The cell death of 5-FU/LDH and siRNA/LDH at 1.2 μg/mL of 5-FU and 40 nM of siRNA were 46% and 34%, respectively, while CD-siRNA-5-FU/LDH complexes showed 70% cell death with the same concentration (Figure 9E). The CD siRNA-5-FU/LDH also significantly decreased a Bcl-2 level, which is related to an anti-apoptotic protein (Figure 9F). Therefore, it was a relatively good example to suppress cancer cell growth via combination therapy.

Combination therapy has been obviously considered a strong strategy to treat cancer with a synergetic effect since two or more therapies are accepted for therapy. Other therapeutic skills such as thermotherapy, photothermal therapy (PTT), photodynamic therapy (PDT), and chemodynamic therapy (CDT) have a high chance of possibility using the LDH system for combination therapy (e.g., chemo/PTT, chemo/PDT, PTT/CDT, etc.) [67,68,69,70,71,72,73,74,75,76,77,78]. For example, the release rate of drugs in LDH nanohybrids is higher when it goes to a higher temperature. In the case of DOX-MgAl-LDHs conjugated with Fe_3_O_4_ (MNHs), thermotherapy and chemotherapy were combined for high drug-loading efficiency and increased rate of drug release [79]. MNHs were found biocompatible for murine fibroblast (L929) and human cervical cancer (HeLa) cell lines. Although it is hard to study which therapy method is well-matched with other skills for combination therapy, repeated attempts must be continued to discover a better biomedical LDH nanosystem.

## 4. Conclusions

Various types of drug delivery vehicles and therapeutic agents were researched for cancer treatment. Among 2D materials, LDH nanohybrids have been expected for promising inorganic materials with biocompatibility to apply various strategies for targeted cancer therapy with potential tumor imaging by intercalation of drugs inside LDH, surface functionalization, and lattice-engineering technique for the inorganic host layers, as described in Table 1. We summarized the therapeutic functions of various LDH nanohybrids with cell lines and/or animal models that are used in vivo and in vitro for proof-of-principle experiments. The physicochemical properties of LDH nanohybrids (facile synthesis, pH-dependent solubility, clathrin-mediated cellular uptake, low toxicity, colloidal stability) allow for the achievement of a drug delivery system and improvement in the utilization of biomedical applications. 

Although we reviewed four different types of therapeutic applications by LDH nanohybrids, namely gene therapy, chemotherapy, immunotherapy, and combination therapy, there will be studies on novel approaches in cancer treatment with higher efficacy that have not been described so far. In the future, we will have a chance to focus on the next step such as clinical trials with FDA approval. For those next steps, comparable studies on other types of drug delivery vehicles should be carried out under the same injection protocols along with reproducible mass production for clinical use. Moreover, the development of new types of LDH nanohybrids will be extended to anti-virial and virus-induced disease treatment that could be one of the potential therapeutic approaches. 

## Figures and Tables

**Table 1:** A brief summary of LDH nanohybrids with cancer therapeutic functions.

LDH Host	Synthetic Method	Biofunctional Molecules	Cell Line and Animal Model	Applications	Key Feature	Refs.
MgAl	Co-precipitation	siRNA	U2OS cells	Gene therapy	Gene therapy with siRNA-based LDH	[28]
MgAl	Ion-exchange	DNA, FITC, adenosine triphosphate	HL-60 cells, NIH3T3 cells	Gene therapy	Gene delivery system with high transfection efficiency	[24]
MgAl	Co-precipitation, silane coupling	siRNA, FITC	KB cells, A549cells, xenograft mice model-bearing KB tumor	Gene therapy, fluorescence imaging	siRNA-based gene therapy in vivo, Selective tumor targeting conjugated with FA	[35]
MnAl	Co-precipitation, self-assembly	siRNA, Mn^2+^	Neuro-2a cells	Gene therapy, MRI	Therapeutic application with T_1_-weighted MRI	[44]
MgAl	Co-precipitation	MTX	orthotopic breast cancer	Chemotherapy	Treated on orthotopic tumor model	[46]
MnFe	Co-precipitation	MTX, Mn^2+^	Hep G2 cells, HeLa cells	Chemotherapy, MRI	First utilization on MnFe-LDH	[54]
MgAl	Co-precipitation, ion-exchange	DOX, PAA	MG-63 cells, A549 cells	Chemotherapy	Boosting the DOX activity with PAA	[56]
NaCa	Urea hydrolysis	Dacarbazine (DAC)	MCF-7 cells, A-375 cells	Chemotherapy	Utilization of NaCa-LDH with DAC	[48]
GdMgAl	Co-precipitation, self-assembly	DOX, Gd^3+^, Au NPs	L929 cells, HeLa cells, mice-bearing 4T1 murine breast tumor	Chemotherapy, MRI, CT	Selective cancer targeting in vivo through EPR effect	[52]
CoMgAl	Co-precipitation, ion-exchange	MTX, Co-57	CT-26 cells	Chemotherapy, SPECT, CT	The in vivo SPECT/CT images with LDH labeled RI	[31]
MgAl	Co-precipitation	CpG, OVA	E.G7-OVA cells	Immunotherapy	Compared to different routes for effective injection	[65]
MgAl	Co-precipitation	OVA	E.G7-OVA cells, female C57BL/6 mice	Immunotherapy	Demonstration of protein-based antitumor vaccine adjuvants	[63]
MgAl	Co-precipitation	miR155	TC-1 murine cervical cells, RAW264.7 murine macrophage-like cells	Immunotherapy	Combinational cancer immunotherapy	[66]
MgAl	Co-precipitation, ion-exchange	5-FU, CD-siRNA	MCF-7 cells, U2OS cells, HCT-116 cells	Combination therapy (Gene, Chemo)	Combined two therapeutic applications.	[34]
